# Simultaneous Detection of Antigen and Antibodies of African Swine Fever in a Novel Combo Lateral Flow Assay

**DOI:** 10.3390/vaccines12030307

**Published:** 2024-03-14

**Authors:** Cristina Aira, Gabriela González-García, Juan Martínez-Cano, Nuria de la Roja, Monica Giammarioli, Francesco Feliziani, Žanete Šteingolde, Jurate Buitkuviene, Petr Václavek, Dimitrije Glišić, Carmina Gallardo, Patricia Sastre, Marga García-Durán, Paloma Rueda, Alba Fresco-Taboada

**Affiliations:** 1 Gold Standard Diagnostics Madrid (GSD Madrid), Calle de los Hermanos García Noblejas 39, 28037 Madrid, Spain; 2 Istituto Zooprofilattico Sperimentale dell’Umbria e delle Marche “Togo Rosati” (IZSUM), Via Salvemini 1, 06126 Perugia, Italy; 3 Institute of Food Safety, Animal Health and Environment “BIOR”, Lejupes Street 3, LV-1076 Riga, Latvia; 4 National Food and Veterinary Risk Assessment Institute, J. Kairiūkščio g. 10, LT-08409 Vilnius, Lithuania; 5 Department of Virology, State Veterinary Institute Jihlava, Rantirovska 93/20, Horni Kosov, 586 01 Jihlava, Czech Republic; 6 Virology Department, Institute of Veterinary Medicine of Serbia, Janisa Janulisa 14, 11000 Belgrade, Serbia; 7 European Union Reference Laboratory for African Swine Fever, Centro de Investigación en Sanidad Animal (CISA-INIA/CSIC), Carretera Algete-El Casar de Talamanca, Km. 8.1, 28130 Madrid, Spain

**Keywords:** African swine fever, antigen detection, antibody detection, rapid test, point of care

## Abstract

African swine fever (ASF) is a contagious disease of wild boar and domestic pigs notifiable to the World Organisation for Animal Health due to its high socio-economic impact. ASF is caused by the complex ASF virus (ASFV), and it can present different clinical manifestations that can be confused with other diseases; for this reason, laboratory testing is necessary for the proper diagnosis of clinically suspected animals. Despite the efforts put into it over decades, no treatment or safe vaccine is globally available, and disease control is based on early diagnosis and the implementation of strict biosecurity measures. In this context, rapid tests have the potential to accelerate and facilitate the identification of infected animals by giving fast on-site results. In this work, we improved the available point-of-care assays for the diagnosis of the disease by the development of a more specific antigen test and a more sensitive antibody test. This antibody detection test allowed for the earlier detection of infected animals than two commercial indirect ELISAs (statistically significant). Moreover, we developed a combined dual rapid test, unifying, in the same cassette, an antigen detection strip and an antibody detection strip. In this study, we confirmed that this combo approach is a useful tool for implementing rapid tests in the field since it increases the percentage of positive samples detected, even when PCR turns negative, while maintaining a good specificity.

## 1. Introduction

African swine fever (ASF) is a contagious disease of wild boar and domestic pigs which threatens animal wellbeing and the swine industry. Due to its high socio-economic impact and its transboundary potential, ASF is a notifiable disease by the World Organisation for Animal Health (WOAH) [1,2]. Since its first description in 1921, the disease has spread around the globe affecting different countries in Europe, America, Africa, and Asia. To date, far from eradication, the disease has been reintroduced in countries which managed to eradicate it decades ago such as Italy, Belgium, Haiti, and the Dominican Republic [3,4,5]. Despite all the efforts put into place, there are no globally available, safe and effective vaccines. The only on-farm trials have been reported in Vietnam [6,7] and in the Dominican Republic. For this reason, ASF control is still based on early diagnosis and on the enforcement of strict sanitary measures [8].

Animal clinical signs are highly influenced by the host’s characteristics and the viral strain which causes the infection, ranging from an almost unapparent disease in the chronic form to a haemorrhagic fever with high fatality rates and ASF-classical symptoms in the acute form of the disease. Different manifestations of the disease make it difficult to diagnose ASF and require the implementation of laboratory testing for the correct identification of the pathogen and for avoiding confusion with other pathogens like Classical Swine Fever Virus [3,9].

The disease is caused by the infection with ASF virus (ASFV), which is a large and complex DNA virus composed of several layers, including two capsids and two lipid membranes. Among the multiple peptides and proteins that form the viral DNA, 54 have been identified as structural proteins [2,10,11,12]. The ASFV genome shows low variation despite its high circulation. A sequence comparison of the *B646L* gene, codifying the viral protein p72, has led to the identification of several viral genotypes due to changes in the carboxyl-terminal end of the protein [13,14,15]. The p72 protein is one of the virion proteins with high antigenic potential; it is highly conserved among genotypes (97.8–100% identity), and it is estimated that this protein comprises around one third of the total proteinic content of the virion, with 8280 copies per particle [1,10]. For these reasons, p72 has long been considered an important target for ASF diagnosis in both PCR-based and serology methods [10,13].

WOAH defines that virus identification can be performed through virus isolation, a fluorescent antibody test, PCR, or antigen detection ELISA. For antibody detection, ELISA, indirect immunoperoxidase test, indirect fluorescent antibody test, and immunoblotting are recommended [8]. All these methods require trained personnel and must be performed in a laboratory. On the contrary, lateral flow assays (LFAs) are point-of-care tests, user-friendly, give rapid results, and exhibit long-term stability over a wide range of climates, making them a useful tool in outbreak scenarios and for the routine testing of animals showing compatible symptoms, especially in low-income countries [16,17]. The combination of antigen and antibody detection methods is particularly relevant for ASF monitoring, not only for animals in the acute phase of the infection but also carrier animals, which play a role in the transmission. Indeed, this combined antigen/antibody detection strategy has been proven efficient in eradicating ASF in the past [18].

For the development of diagnostic tests, the production of recombinant proteins offers several advantages over the production of native proteins. The production of recombinant antigens effectively minimizes the challenges associated with handling infectious agents. This method not only yields greater quantities but also simplifies standardization and scaling up processes. Additionally, it demonstrates minimal variations in epitopes [19,20]. Moreover, the use of recombinant monoclonal antibodies allows a higher characterization of these molecules, it enables the production of more reproducible batches, and it opens the possibility of engineering these proteins [21,22].

In this work, we describe the development of new point-of-care tools for the control of ASFV with improved performance: (1) an antigen detection test based on a recombinant antibody against p72; (2) an antibody detection test based on a recombinant form of the p72; (3) a Combo test combining the two assays in the same cassette for the parallel detection of an antigen and antibody in a given sample.

## 2. Materials and Methods

### 2.1. Blood and Serum Samples

In this study, different blood and serum panels collected from the field or from experimental studies were employed. This panel is described in Table 1. For antigen detection, the reference technique used was the WOAH real-time PCR, while for antibody detection the reference technique used was IPT. Whenever IPT results were not available, a competitive ELiSA (INgezim^®^ PPA Compac, Madrid, Spain) was performed to properly characterize the samples.

### 2.2. Recombinant Proteins Production

Recombinant antibody production was carried out as previously described [23]. The recombinant p72 was expressed in mammal cells (FreeStyle™ 293-F Cells (Invitrogen, Waltham, MA, USA)) by plasmid transfection and purified from a soluble fraction by affinity chromatography.

### 2.3. Lateral Flow Assays

For the antigen detection in blood samples, a double antibody sandwich assay employing latex nanoparticles was developed (INgezim^®^ ASFV CROM Ag 2.0, GSD Madrid, Madrid, Spain). For antibody detection in blood or serum samples, a double recognition assay (or double antigen sandwich assay) based on colloidal gold nanoparticles was optimized (INgezim^®^ ASFV CROM Ab 2.0, GSD Madrid, Madrid, Spain).

#### 2.3.1. Capture Reagents

The commercial monoclonal antibody 18BG3 (GSD Madrid, Madrid, Spain), specific to the p72 of ASFV, was diluted in 20 mM Tris-HCl pH 7.5 buffer containing sucrose and used as test line reagent for the antigen detection test. The recombinant form of the p72 was diluted in Tris-HCl pH 8.5 containing sucrose and was used as a test line reagent for the antibody detection test. An antibody specific to the control protein (U-10 MAb; Operon, Zaragoza, Spain) was diluted in 20 mM Tris-HCl pH 7.5 buffer containing sucrose, and the solution was used as control line reagent in both assays.

The described mixtures were dispensed in parallel (one test and one control line) onto nitrocellulose membranes. The resulting membrane was dried for 5 min at 45 °C and stored at room temperature under dry conditions.

#### 2.3.2. Detector Reagents

**Figure 1:**
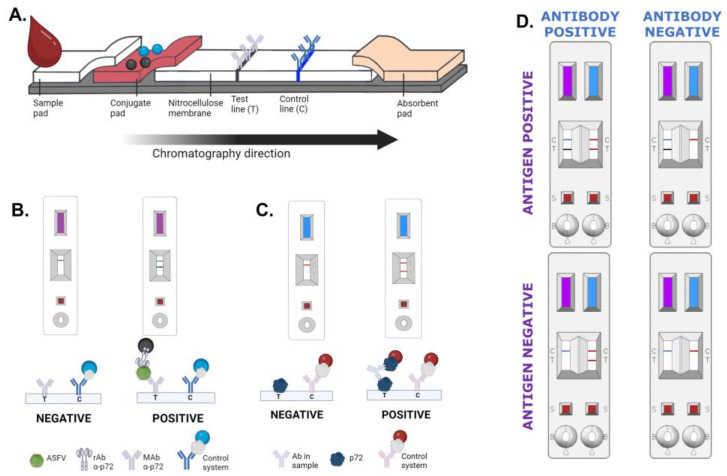
(**A**) General scheme of the lateral flow strip. (**B**) Result interpretation for the INgezim^®^ ASFV CROM Ag 2.0, purple strip. (**C**) Result interpretation for INgezim^®^ ASFV CROM Ab 2.0, blue strip. (**D**) Result interpretation for INgezim^®^ ASFV Combo CROM Ag/Ab (GSD Madrid, Madrid, Spain). Created with Biorender.com.

For the antigen detection test, the recombinant scFv-Fc-18BG3 antibody and the control protein were covalently coupled to carboxylated polystyrene black (Merck, Darmstadt, Germany) or blue (Ikerlat, Gipuzkoa, Spain) latex nanoparticles, respectively. Briefly, carboxyl groups on the surface of the nanoparticles were activated according to the EDC/NHS protocol [24]. Then, activated nanoparticles were separately incubated with the different reagents, which were added to obtain a final concentration of 1 mg/m^2^. After blocking the non-reacted functional groups, particles were diluted to 1% (*w*/*v*) in 10 mM Tris-HCl pH 8.2 and they were stored at 4 °C (Figure 1B). To prepare the conjugate solution, the scFv-Fc-18BG3 black latex was mixed with control blue latex diluted to final concentrations of 0.1% (*w*/*v*) and 0.15% (*w*/*v*), respectively. The mixture was dispensed onto the conjugate pad, and the pad was dried for 30 min at 45 °C and stored at room temperature under dry conditions (Figure 1C).

For the antibody detection test, the recombinant form of the p72 and the control protein were coupled to colloidal gold nanoparticles (Nanoflow, Liège, Belgium). Briefly, colloidal gold nanoparticles were diluted to 1 Optical Density at 530 nm (OD_530_) in the selected buffer, next, the proteins were diluted in that same buffer and added to the gold. The coupling reaction was incubated for 1 h at room temperature under stirring. The colloidal gold coupled to p72 was mixed with control particles to a final concentration of 5 OD_530_ and 2.5 OD_530_, respectively, and dispensed onto a conjugate pad. The pads were dried for 30 min at 45 °C and stored at room temperature under dry conditions.

#### 2.3.3. Assembling of LFA Strips

The nitrocellulose membrane was pasted on an adhesive card (backing card), followed by conjugate and absorbent pads, both overlapping the nitrocellulose membrane. The sample pad was pasted on the backing card overlapping the conjugate pad. Finally, an adhesive cover tape was pasted onto the sample pad/conjugate pad/membrane interface (Figure 1A). This master card was cut into 4.2 mm width strips, and individual strips were assembled into cassettes. For the INgezim^®^ ASFV Combo CROM Ag/Ab, an individual strip for antigen detection and an individual strip for antibody detection were both assembled into a cassette, as shown in Figure 1D. The cassettes were stored at room temperature in aluminium foils under dry conditions.

#### 2.3.4. Test Procedure

For the analysis of blood samples (antigen and antibody detection assays), 20 µL of blood was added to the window labelled as “S”. After blood absorption, 5 drops (150 µL approx.) of the corresponding running buffer were added to the window labelled as “B”. Results were read after 15 min of buffer addition by the naked eye.

For the analysis of serum samples (antibody detection assay), 10 µL of serum was added to the window labelled as “S”. After its absorption, 5 drops (150 µL approx.) of the corresponding running buffer were added to the window labelled as “B”. The results were read by the naked eye after 15 min of buffer addition.

### 2.4. Statistical Analysis

The 95% confidence intervals (CIs) were calculated for the different groups of samples established (ASFV samples classified according to days post-infection or PCR cycle Cycle quantification (Cq) value). The statistical significance between techniques was determined for each group of samples by a McNemar test employing the open-access software OpenEpi version 3.01 [25].

Specificity (Sp) was calculated as follows:

Specificity = (True negative)/(True negative + False positive) × 100(1)

Samples characterised as negative, which gave a positive result with the developed assays, were considered “false positive” results, while concordant negative results were considered “true negative”.

## 3. Results

### 3.1. Antigen Detection Assay: INgezim^®^ ASFV CROM Ag 2.0

**Figure 2:**
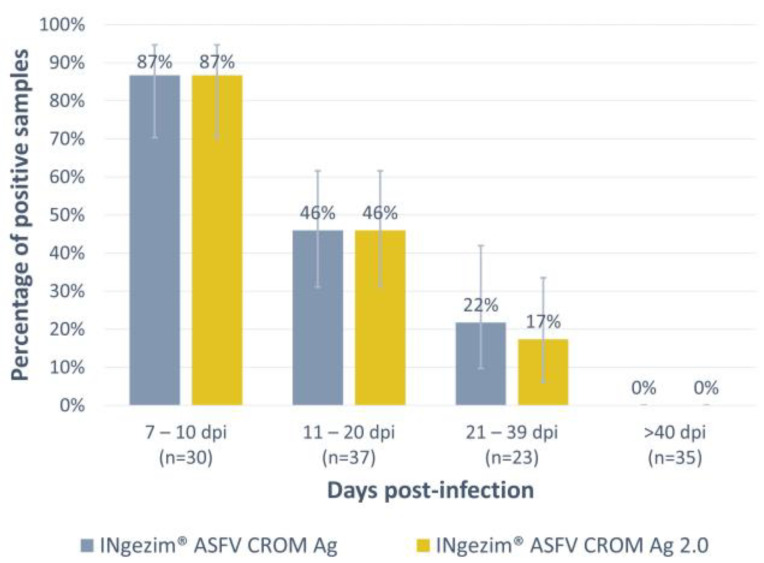
Analysis of experimental blood samples positive to ASFV antigen by the commercial test INgezim^®^ ASFV CROM Ag and the new version of the assay developed: INgezim^®^ ASFV CROM Ag 2.0. The *X*-axis shows the four different groups tested according to days post-infection. The *Y*-axis shows the percentage of positive samples found per group. Error bars show the 95% confidence interval for each group.

The assay was optimized and then evaluated with a total of 125 experimental blood samples, all of them positive based on the WOAH real-time PCR (Table 1, panel 1). As shown in Figure 2, antigen detection was higher during the first days of infection, and it gradually decreased over time, not detecting positive samples above 40 days post-infection (dpi). The detection rate observed with the new version of the test, INgezim^®^ ASFV CROM Ag 2.0, was comparable to the commercially available test used for comparison (INgezim^®^ ASFV CROM Ag, GSD Madrid, Madrid, Spain). All the groups tested exhibited the same percentage of positive samples with both tests except for the group of 21–39 dpi, in which this percentage was slightly lower with the new version of the test (one positive sample detected as negative). However, no statistically significant difference was observed in any of the groups tested.

Specificity (Sp) was evaluated with a total of 315 negative field samples collected from free (Table 1, panel 2) or endemic areas (Table 1, panel 3). All samples were considered as real negative samples from the results obtained combining both virus and antibody detection reference tests. The newly developed assay exhibited an improved specificity compared to the commercially available test. For the samples in panel 2, the commercial assay exhibited a 98% Sp, while the new assay showed a Sp of 100%. Moreover, in samples from panel 3, in which the commercial assay showed a low specificity (92.0%), the new version of the test exhibited an improved specificity of 98.7%. The overall specificity of the newly developed test was 99.4%, with only two false positive results among the 315 samples tested.

### 3.2. Antibody Detection Test: INgezim^®^ ASFV CROM Ab 2.0

**Figure 3:**
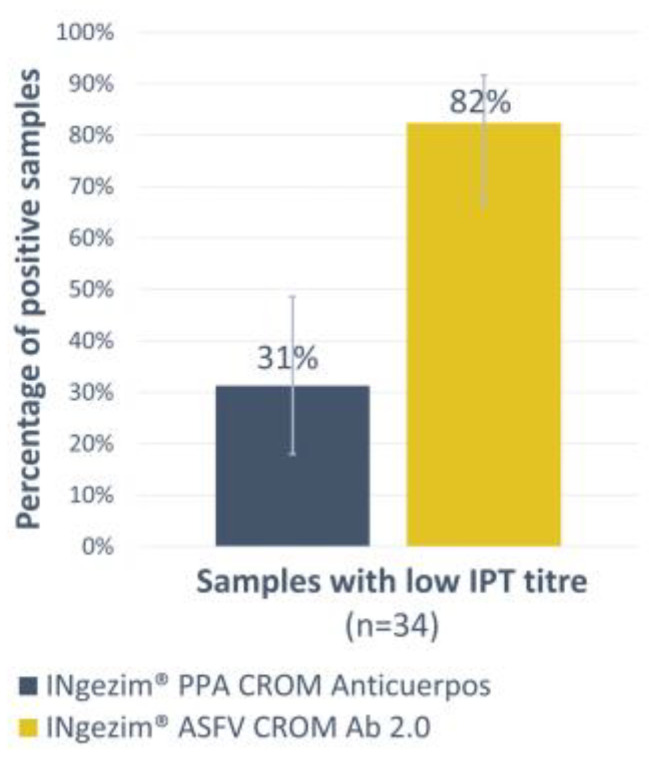
Low antibody titre sera analysis by INgezim^®^ PPA CROM Anticuerpos and INgezim^®^ ASFV CROM Ab 2.0. The *Y*-axis shows the percentage of positive samples. Error bars show the 95% confidence interval for each group.

For the antibody detection, a double recognition assay was developed. First, the new assay was compared with a previous commercial lateral flow test designed with the same format, INgezim^®^ PPA CROM Anticuerpos (GSD Madrid, Madrid, Spain). Thirty-four experimental sera with low antibody titres, as measured by the reference technique IPT (Table 1, panel 4), were analysed using both tests. While the INgezim^®^ PPA CROM Anticuerpos only detected 31% of the samples as positive, the new version increased that percentage to 82% (Figure 3). A high statistical significance between both tests was observed with a *p*-value of 0.0001.

Considering that ELISA tests are widely utilized in laboratory settings, the sensitivity of the new test was assessed in comparison to two other techniques, specifically two indirect commercial ELISAs recognized for their superior sensitivity compared to rapid tests. These two assays were based on distinct African Swine Fever Virus (ASFV) proteins: the INgezim^®^ ASFV-R utilizing p30 and CP312 antigens (GSD Madrid, Madrid, Spain) and the ID Screen^®^ African Swine Fever Indirect relying on p32, p62, and p72 antigens (IDvet, Grabels, France). To conduct this evaluation, a set of 92 positive experimental sera was analysed (Table 1, panel 5).

**Figure 4:**
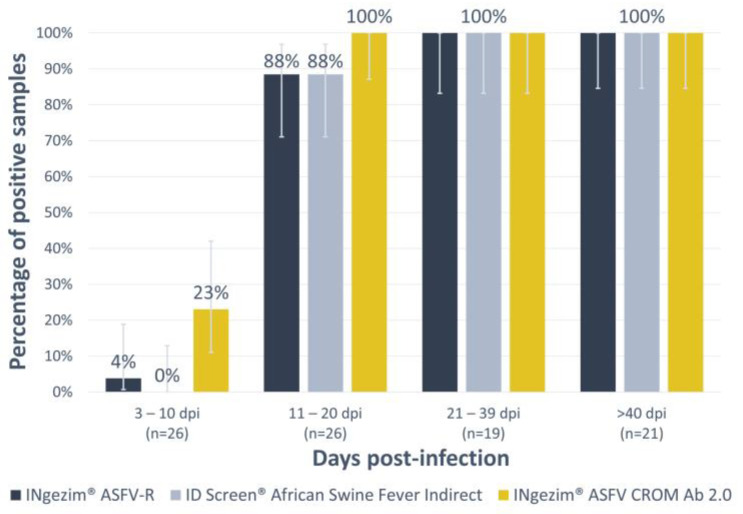
Analysis of experimental sera with the new assay INgezim^®^ ASFV CROM Ab 2.0 and with the indirect ELISAs INgezim^®^ ASFV-R and ID Screen^®^ African swine fever Indirect. The *X*-axis shows the different groups according to days post-infection (dpi). The *Y*-axis shows the percentage of positive samples. Error bars show the 95% confidence interval per group and assay.

As depicted in Figure 4, it is evident that in all instances, the proportion of positive samples increases steadily over time up to 56 dpi (last point screened). The new lateral flow assay demonstrated a higher level of sensitivity compared to the indirect ELISAs when it came to detecting antibodies at earlier stages. This difference was statistically significant, particularly in the 3 to 10 days post-infection group, where it yielded a *p*-value of 0.02 against the ID Screen^®^ African Swine Fever Indirect and a *p*-value of 0.04 against the INgezim^®^ ASFV-R. In the 11 to 20 days post-infection group, the differences were less pronounced, with a *p*-value of 0.13 against both indirect ELISAs.

The specificity of the new assay was evaluated with a collection of 296 serum samples from field or from experimental studies which were positive to other pig diseases (Table 1, panels 6–9). The assay exhibited a specificity of 99.6%, finding one false positive result among field-negative sera, and another false positive result in the group of wild boar samples positive for antibodies to tuberculosis.

### 3.3. Combined Antigen and Antibody Detection, INgezim^®^ ASFV Combo CROM Ag/Ab

**Figure 5:**
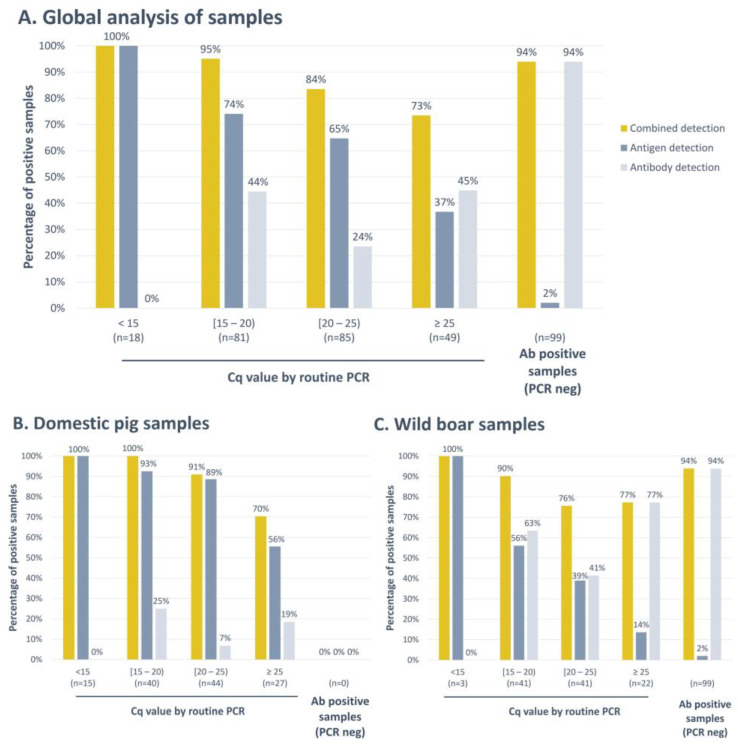
Analysis of field blood samples with the INgezim^®^ ASFV Combo CROM Ag/Ab assay (**A**). (**B**) Result obtained only for domestic pig samples. (**C**) Result obtained only for wild boar samples. Combined detection results show the percentage of positive samples by any of the strips included in the cassette, antigen, or antibody. Antigen detection shows percentages of positive results obtained only with the antigen detection strip. Antibody detection shows percentages of positive results obtained only with the antibody detection strip. The *X*-axis shows the groups of samples divided according to their viral load by PCR (Cq). The *Y*-axis shows the percentage of positive samples in each group.

As seen in Figure 2 and Figure 4, the antigen detection assays exhibited high detection rates in the early days post-infection with marked reductions as the infection developed, while antibody detection increased with days post-infection. For this reason, once individual assays were validated, a novel assay, INgezim^®^ ASFV Combo CROM Ag/Ab, was developed, combining both strips. To analyse the sensitivity of this combined test, a group of 332 field positive samples (Table 1, panel 10) was evaluated. These samples were grouped by their ASFV genome load using PCR Cq values as a reference. The samples which were negative by PCR but positive by serology were included in the fifth group “Ab positive samples (PCR neg)” (Figure 5).

As depicted in Figure 5A, when analysing samples with Cq values below 15, the antigen detection strip consistently yielded positive results, detecting 100% of these samples as positive. Conversely, the antibody detection strip did not yield any positive results under these conditions. As the ASFV genome content decreased, a corresponding reduction in the number of samples testing positive for the viral antigen was observed. In these instances, the antibody detection strip played a complementary role in the detection of infected animals. This transition was clearly evident, with the percentage of positive samples increasing from 74% to 95% within the Cq range of 15 to 20, from 65% to 84% in the Cq range of 20 to 25, and having a substantial rise from only 37% positive samples to 73% in the group with Cq values greater than 25.

Furthermore, when PCR failed to detect an infected animal, serological testing demonstrated its effectiveness by identifying 99 positive animals. Among these, the new Combo rapid test successfully detected 94% of them as positive (Figure 5A). The new Combo assay showed a global sensitivity of 88.9%.

Among these results, differences were obtained with samples collected from domestic pigs and those obtained from wild boar. As shown in Figure 5B, when separately analysing the samples collected from domestic pigs, we observed that no samples characterised as positive by serology and negative by PCR were collected during the surveillance campaigns. The identification of infected animals was mainly performed via antigen detection, and antibody detection slightly improved the percentage of positive samples, passing from a total of 84.1% of positive samples detected with the antigen detection strip to 90.5% with the Combo test. On the other hand, when analysing wild boar samples separately (Figure 5C), we observed that the percentage of positive samples identified by the antigen detection strip was lower and that, in this case, the combined detection of antigens and antibodies improved the identification of infected animals on-site. The percentage of positive samples detected increased from 56% to 90% in the group of samples with a Cq value in PCR between 15 and 20; from 39% to 76% in the group of Cq between 20 and 25; and from only 14% to 77% in samples with a Cq greater than 25. Moreover, all the samples characterised as negative by PCR and positive by serology were collected from wild boar. In this species, the Combo test exhibited a global sensitivity of 87.9%.

Moreover, to determine the assay’s specificity, a group of 193 negative field samples were tested (Table 1, panel 11). The combined antigen and antibody detection assay exhibited a global specificity of 97.4%, 98.0% for samples collected from domestic pigs, and 96.8% for samples collected from wild boar. Three samples collected from wild boar and two samples collected from domestic pigs gave a false positive result. All these false positive samples were detected by the antigen detection strip.

## 4. Discussion

ASF is an infectious disease which, nowadays, is strongly affecting swine production around the globe, with Asia, Africa, Eastern Europe, and the Indo-Pacific regions being markedly affected [5]. To date, no treatment has been described, and although some vaccines are undergoing field investigations, they are not globally available. The control of this pathogen is still based on the early detection of infected animals [26,27], for which a laboratory tool is crucial due to the complex clinical syndrome of the disease [3,8,9]. In this context, rapid tests have the potential to accelerate the final identification of affected animals by the on-site detection of the disease without the need for highly qualified staff or equipment, and they are easy to use and can be read by the naked eye [16,17].

In this work, we have improved the already available point-of-care tools for the on-site detection of ASFV-infected animals. On the one hand, the use of a recombinant antibody allowed the development of a more specific antigen detection test in which specificity was shown to increase from 92.0% to 99.4%, maintaining the same sensitivity as the commercial assay. On the other hand, we have developed a more sensitive antibody detection test, which is able to detect lower antibody titres in samples characterized as weak positive by IPT, and which shows the potential to detect infection slightly earlier than the indirect ELISAs used for comparison.

In the context of ASF diagnosis, it is important to note that both viral antigen and antibody detection tests show a significant time-dependent pattern. In these terms, our study yielded noteworthy observations compliant with previous works [28]. When the viral load was elevated, the antigen detection strip reliably identified infections, with no concurrent presence of antibodies. In contrast, as the viral load decreased, the implementation of the combined test demonstrated enhanced efficacy, resulting in an increased percentage of positive samples across all tested groups. Notably, when no viral load was detected, the antibody detection strip allowed the identification of 93 wild boar samples which would have been considered negative if only analysing samples via an antigen detection strip. This finding is of particular interest since animals that have survived infection or have been infected with lower virulence strains, and which can consequently be detected at later days post-infection, can play a role in ASFV transmission and should be considered to control the disease [8]. The Combo cassette described here that combines both strips, for antigen and antibody detection, offers a great advantage in these scenarios, allowing the detection of an antigen and antibody simultaneously in a given sample. It is worth mentioning that this combined surveillance strategy has been employed in the past for the eradication of African Swine Fever Virus (ASFV) in certain countries, and it was shown to yield favourable outcomes [18].

The results presented in this study indicated that the new Combo assay can be used with samples collected from wild boar as well as from domestic pigs, giving good results by both antigen and antibody detection strips. This finding indicates that the developed assays could be applied to the surveillance of both species equally, although greater improvement is observed for wild boar samples. This different behaviour with wild boar samples could be explained by several factors. The sample quality: for wild boar surveillance, carcasses are often used, and the blood collected is usually clotted; this sample quality can lead to lower viral detection. The clinical control: symptoms are only tracked in domestic pigs, which turns into a faster identification of infection and to the collection of samples in the early days post-infection in which animals have high viral loads in blood and no antibodies yet. Further studies using experimental wild boar samples will be carried out to further confirm these findings.

In conclusion, in this study, we improved the available tools for ASF antigen and antibody detection, and we developed the first diagnostic tool for the combined Ag/Ab detection of ASF. The new rapid tests developed showed an ability to facilitate the on-site diagnosis of ASF, proving to be potential tools for both the confirmatory diagnosis of animals with compatible signs and able to help in field surveillance and outbreak investigations. While antigen detection directly indicates an active infection, antibody detection should be considered together with other clinical/epidemiological information to properly identify the clinical implications of the diagnosis.

## Figures and Tables

**Table 1:** Summary of the sample collections used in the present study.

Collection	Type of Sample	Number of Samples	ASFV Status (Technique Used)	Origin	Collection Details
Panel 1	Blood	125	Antigen positive (WOAH real-time PCR)	Experimental (CISA-INIA)	Domestic pigs experimentally inoculated with different ASFV strains, including genotypes I (*n* = 11), II, (*n* = 42), I–II (*n* = 1), IX (*n* = 1), and XXIII (*n* = 1), in biosafety level 3 (BSL3) facilities.
Panel 2	Blood	165	Negative	Field	Field pig samples collected from Spanish farms (ASFV-free region).
Panel 3	Blood	150	Negative	Field	Negative samples collected from endemic areas from healthy pigs, characterized as negative by PCR and ELISA.
Panel 4	Serum	34	Antibody positive (IPT)	Experimental (CISA, INIA-CSIC)	Domestic pig samples collected from experimental infections carried out in BSL3 facilities and classified as positive by IPT with low antibody titres.
Panel 5	Serum	92	Antibody positive and negative (ELISA)	Experimental (IZSUM)	Samples collected from 9 different domestic pigs experimentally infected with the attenuated ASFV strain NH/P68 in BSL3 facilities.
Panel 6	Serum	208	Negative	Field	Domestic pig samples collected from Spanish farms (ASFV-free region).
Panel 7	Serum	47	Negative	Field	Field wild boar sera characterized as positive to tuberculosis (TB) collected in Spain.
Panel 8	Serum	26	Negative	Field	Pig sera positive to Porcine Respiratory and Reproductive Virus specific antibodies (PRRSV) collected in Spanish farms.
Panel 9	Serum	15	Negative	Experimental (FLI)	Pig sera positive to Classical Swine Fever Virus (CSFV), Border Disease Virus (BDV), or Bovine Viral Diarrhoea Virus (BVDV) specific antibodies.
Panel 10	Blood	332	Antigen positive (PCR) and/or antibody positive (ELISA or IPT)	Field	Field samples collected during outbreak investigations in Latvia, Lithuania, Czech Republic, and Republic of Serbia: 126 domestic pig and 206 wild boar samples.
Panel 11	Blood	193	Negative (PCR and/or IPT and/or ELISA)	Field	Field samples collected during outbreak investigations in Latvia, Lithuania, Czech Republic, and Republic of Serbia: 100 domestic pig and 93 wild boar samples.

IPT: Immunoperoxidase test. CISA, INIA-CSIC: Centro de Investigación en Sanidad Animal. IZSUM: Istituto Zooprofilattico Sperimentale dell’Umbria e delle Marche. FLI: Friedrich-Loeffler Institute.

## Data Availability

The data presented in this study are available on request from the corresponding author.
